# Prevalence and characteristics of anxiety in patients with unconfirmed pulmonary nodules

**DOI:** 10.1111/crj.13576

**Published:** 2023-01-18

**Authors:** Xiao‐Hui Wang, Ting Wang, Min Ao, Jinglan He, Jun Duan, Long‐Biao Cui, Shuliang Guo, Li Yang

**Affiliations:** ^1^ Department of Respiratory and Critical Care Medicine The First Affiliated Hospital of Chongqing Medical University Chongqing P. R. China; ^2^ Department of Psychiatry The First Affiliated Hospital of Chongqing Medical University Chongqing P. R. China; ^3^ Department of Clinical Psychology Fourth Military Medical University Xi'an P. R. China

**Keywords:** anxiety, characteristic, prevalence, pulmonary nodules

## Abstract

This study focuses on the prevalence and characteristics of anxiety in patients with pulmonary nodules that was assessed by Hamilton Anxiety Scale (HAMA) scores. A total of 890 patients were enrolled in this study, including incidence of absence of anxiety *n* = 343 (38.54%), mild or probable anxiety *n* = 459 (51.57%) and moderate or definite anxiety *n* = 79 (8.88%) and obvious anxiety *n* = 9 (1.01%), respectively. According to the definition of anxiety, 88 (9.89%) patients were enrolled in anxiety group. The incidence of anxiety in females was significantly higher than male (11.98% vs. 7.20%, *p* = 0.018), patients with respiratory symptoms were significantly higher than without respiratory symptoms (13.33% vs. 8.50%, *p* = 0.029) and diameter of pulmonary nodules >8 mm is significantly higher than ≤8 mm (13.35% vs. 7.10%, *p* = 0.002). Regression analysis showed that female (OR = 0.548, 95% CI: 0.340–0.884), family history of malignant tumour (OR = 1.691, 95% CI: 1.067–2.678), respiratory symptoms (OR = 1.713, 95% CI: 1.073–2.733) and diameter >8 mm (OR = 2.135, 95% CI: 1.350–3.375) were independent risk factors of anxiety. Further analysis of 88 patients with anxiety showed the sum of psychic anxiety was significantly higher than somatic anxiety (16.66 ± 2.46 vs. 0.97 ± 1.10, *p <* 0.0001). Hence, vast majority of patients with unconfirmed pulmonary nodules suffered various severity of anxiety and manifested as psychic anxiety. And gender, respiratory symptoms, family history of malignant tumour and diameter of pulmonary nodules were independent influencing factors of anxiety. Effective strategies urgently need exploring and providing for improving the mental health.

## Graphical abstract

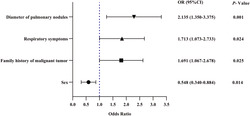

## 1 INTRODUCTION

Lung cancer is one of most common cancers and currently the leading cause of cancer‐related death, accounting for nearly 20%. Moreover, lung cancer mortality in China is relatively high compared to most countries.^1^ Smoking,^2^ air pollution,^3^ and occupational exposure, mainly including asbestos and dust,^4^ have been classified as primary carcinogen. And China is the biggest consumer of tobacco products.^5^ Early detection, diagnosis and treatment are the most effective ways to reduce the mortality. The National Lung Screening Trial (NLST) showed that screening with low‐dose helical computed tomography (LDCT) reduced mortality from lung cancer.^6^, ^7^ And LDCT could help detect early‐stage lung cancer. Fan et al. reported, for the LDCT screening, the positive rate is 29.89%, and the proportion of Stage I of lung cancer detected by LDCT was 81.09%.^8^ Hence, annual lung cancer screening with LDCT is recommended for high‐risk individuals aged 50–74 years who have at least a 20 pack‐year smoking history and who currently smoke or have quit within the past 5 years in China.^9^

However, the proportion of detected lung cancer in all lung nodules ranges from 0.33% to 3.48%.^8^, ^10^, ^11^ In short, the vast majority of lung nodules most are benign, and this part of patients do not benefit from screening of lung nodule. Instead, they are left in an uncertainty state about the ‘near lung cancer’ diagnosis, which cause a huge psychological harm undergo the radiographic surveillance recommended by the expert guidelines for months or years,^9^ as it is difficult and even a great challenge to determine if a nodule is malignant or benign when first identified. Freiman's research indicated approximately 44% anxiety or depression, and 26% clinically significant distress related to their pulmonary nodule, with multiple concerns, including uncertainty about the nodule's cause, the possibility of cancer and the possible need for surgery.^12^ Overall, there was no correlation between perception of lung cancer and actual risk.^12^ In China, Li et al. reported the incidence of anxiety in patients with pulmonary nodules was 59.3% (108/182) via generalized anxiety disorder scale‐7 (GAD‐7) and was affected by social support and previous psychological factors.^13^ And Wang et al. reported 39.8% (41/103) patients with pulmonary nodules were prone to varying degrees of anxiety and depression, leading to immune dysfunction and low‐grade inflammation.^14^ However, another study showed computed tomography (CT) scan did not appear to result in adverse psychological responses compared to those with a normal CT scan.^15^ Gareen et al. reported participants receiving a false‐positive result experienced no significant difference in health‐related quality of life (HRQoL) or state anxiety at 1 or at 6 months after screening relative to those receiving a negative result.^16^ Hence, there are controversies on the psychological harm of pulmonary nodules, and the anxiety's risk factors and characteristic of pulmonary nodules still need to be clarified by a larger sample.

## 2 METHODS

### 2.1 Patients

This was a cross‐sectional study. From January 2019 to August 2020, 890 patients with unconfirmed pulmonary nodules visited the Department of Respiratory and Critical Care Medicine, The First Affiliated Hospital of Chongqing Medical University. All patients have completed medical records and provided written informed consent and then were investigated via demographic and medical characteristics and Hamilton Anxiety Scale (HAMA) scores. Unconfirmed pulmonary nodules were defined as the diameter of pulmonary nodules was ≤3 cm, which had not been confirmed as benign or malignant. Exclusion criterion included age <18‐year‐old, severe cognitive or physical impairment and language communication disorder. The testers were trained in advance to master unified instructions and procedures and ensure the quality of the investigation.

### 2.2 Questionnaire

The investigation consisted of two parts. The first part contained patient's demographic and clinical data, such as gender, age, family history of malignant tumour, second‐hand smoke exposure, history of smoking, profession, history of occupational exposure, respiratory symptom, number of pulmonary nodules, diameter of pulmonary nodules and time since the first discovery. The second part was to evaluate the anxiety state by HAMA, which has been wildly used to assess the severity of anxiety symptoms.^17^, ^18^ The HAMA is a 14‐item clinician‐administered rating scale that measures the severity of anxiety based on the frequency and impairment of symptoms during the past week. Each item ranges from 0 (not present) to 4 (very severe). Higher scores indicate a greater degree of anxiety. Items 7–13, known as the somatic anxiety subscore, sum of which indicate the severity of somatic anxiety, and items 1–6 and 14, known as the psychic anxiety subscore, sum of which indicate the severity of psychic anxiety. Anxiety was evaluated by the sum of HAMA, with a score ≤7 as absence of anxiety, 8–14 as mild or probable anxiety, 15–21 as moderate or definite anxiety, 22–29 as obvious anxiety and more than 29 as severe anxiety. Usually, the anxiety group is defined by sum score >14, and non‐anxiety is ≤14.

### 2.3 Statistical analysis

The data were analysed by SPSS 22. The chi‐square test or Fisher's exact probability method was used to analyse the counting data. The multivariate logistic regression was employed to determine independent risk factors of anxiety. The quantitative data that were not normally distributed were presented as median and quartile [M, (Q1, Q3)], and the Mann–Whitney *U* test was used to determine the differences between two groups. Values were expressed as mean ± standard deviation (SD). *p*‐Value <0.05 was deemed to be statistically significant. Those factors moderately associated with anxiety in bivariate analyses (*p* < 0.1) were included in the multivariable model.

## 3 RESULTS

### 3.1 The distribution of anxiety state according to HAMA scores

**FIGURE 1:**
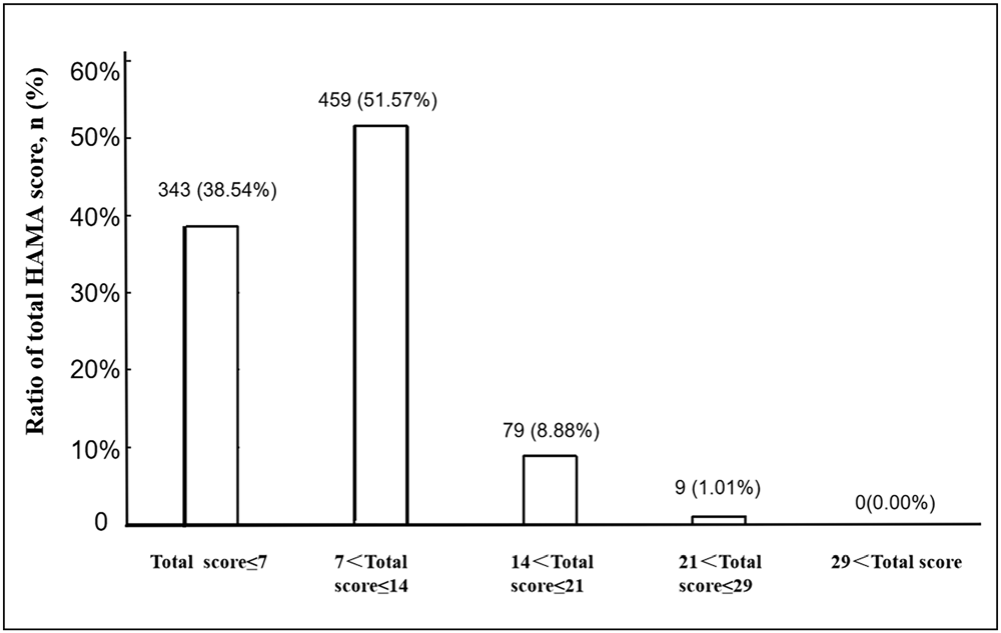
The distribution of HAMA scores in patients with pulmonary nodules

A total of 890 patients with complete medical records and evaluation by HAMA were enrolled in this study, including 343 (38.54%) patients with a score ≤7 as absence of anxiety, 459 (51.57%) patients with a score between 8 and 14 as mild or probable anxiety, 79 (8.88%) patients with a score between 15 and 21 as moderate or definite anxiety, 9 (1.01%) patients with a score between 22 and 29 as obvious anxiety and 0 (0.00%) patients with a score more than 29 as severe anxiety (Figure 1).

### 3.2 The analysis of anxiety impact factors in patients with pulmonary nodules

Among the 890 patients, 88 (9.89%) patients were enrolled in anxiety group and 802 (90.11%) in non‐anxiety group. The anxiety ratio of females is significantly higher than male (11.98% vs. 7.20%, *p* = 0.018), patients with respiratory symptoms was significantly higher than those without respiratory symptoms (13.33% vs. 8.50%, *p* = 0.029) and diameter of pulmonary nodules >8 mm is significantly higher than ≤8 mm (13.35% vs. 7.10%, *p* = 0.002). And there was no significant difference among age, family history of malignant tumour, second‐hand smoke exposure, history of smoking, profession, history of occupational exposure, number of pulmonary nodules or time since the first discovery (Table 1).

**TABLE 1:** Analysis of anxiety impact factors of HAMA scores in patients with pulmonary nodules

		HAMA>14	HAMA≤14	*p*‐Value
Gender				
	Female, *n* (%)	60(11.98%)	441(88.02%)	0.018
	Man, *n* (%)	28(7.20%)	361(92.8%)	
Age				
	≥40‐year‐old, *n* (%)	74(9.48%)	707(90.52%)	0.27
	<40‐year‐old, *n* (%)	14(12.84%)	95(87.16%)	
Family history of malignant tumour				
	Yes, *n* (%)	38(12.50%)	266(87.50%)	0.06
	No, *n* (%)	50(8.53%)	536(91.47%)	
Second‐hand smoke exposure				
	Yes, *n* (%)	65(10.82%)	536(89.18%)	0.181
	No, *n* (%)	23(7.96%)	266(92.04%)	
History of smoking				
	Yes, *n* (%)	16(6.87%)	217(93.13%)	0.072
	No, *n* (%)	72(10.96%)	585(89.04%)	
Profession				
	Yes, *n* (%)	85(9.78%)	784(90.22%)	0.754
	No, *n* (%)	3(14.29%)	18(85.71%)	
History of occupational exposure				
	Yes, *n* (%)	2(4.55%)	42(95.45%)	0.338
	No, *n* (%)	86(10.17%)	760(89.83%)	
Respiratory symptoms				
	Yes, *n* (%)	34(13.33%)	221(86.67%)	0.029
	No, *n* (%)	54(8.50%)	581(91.50%)	
Number of pulmonary nodules				
	Single, *n* (%)	37(9.05%)	372(90.95%)	0.438
	Multiple, *n* (%)	51(10.60%)	430(89.40%)	
Diameter of pulmonary nodules				
	≤8 mm, *n* (%)	35(7.10%)	458(92.90%)	0.002
	>8 mm, *n* (%)	53(13.35%)	344(86.85%)	
Time since the first discovery				
	≤1 month, *n* (%)	77(9.73%)	714(90.27%)	0.665
	>1 month, *n* (%)	11(11.11%)	88(88.89%)	

### 3.3 The analysis of independent influencing factors for anxiety in patients with pulmonary nodules

**FIGURE 2:**
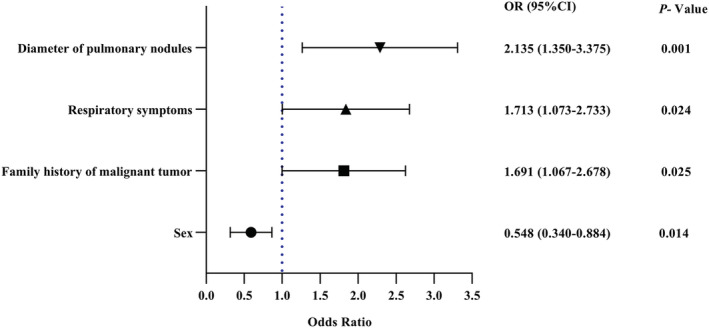
Forward logistic regression analysis of influencing factors of HAMA scores in patients with pulmonary nodules

We brought in five factors with *p*‐Value less than 0.1 in Table 1 for multivariate logistic analysis, including gender, family history of malignant tumour, history of smoking, respiratory symptoms and diameter of pulmonary nodules. Further regression analysis showed that female, family history of malignant tumour, respiratory symptoms and diameter of pulmonary nodule >8 mm were independent risk factors for anxiety in patients with unconfirmed pulmonary nodules (Figure 2).

### 3.4 The analysis of anxiety characteristics in patients with pulmonary nodules

**FIGURE 3:**
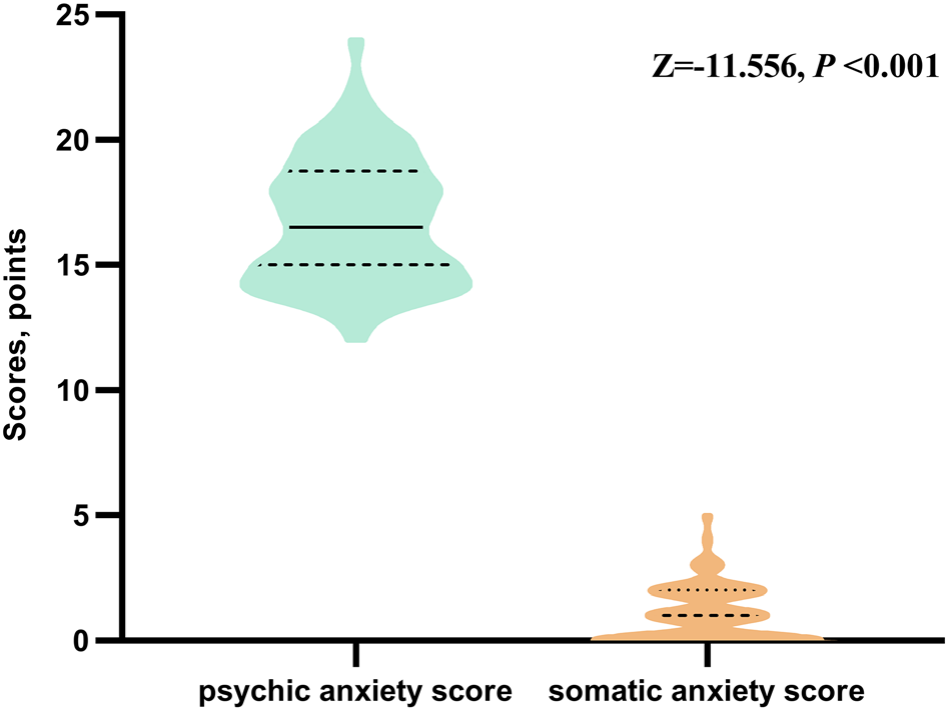
Compare of psychic and somatic anxiety in 88 patients with anxiety

We analysed the differences of each item in HAMA and sum of psychic and somatic anxiety between the anxiety and non‐anxiety group. In the anxiety group, the score of item 1, 2, 3, 4, 6, 7, 8, 10, 13, 14 and sum of psychic and somatic anxiety was significantly higher than non‐anxiety. There was no statistical difference of item 5 (cognitive), item 9 (cardiovascular symptoms), item 11 (gastrointestinal symptoms), item 12, (genitourinary symptoms) and item 13 (autonomic symptoms) (Table 2). Further analysis of 88 patients with anxiety showed the sum of psychic anxiety was significantly higher than somatic anxiety [M, (Q1, Q3); 16.50, (15.50, 18.75) vs. 1, (0, 2), *p* < 0.001] (Figure 3).

**TABLE 2:** Analysis of items of HAMA scores in patients with pulmonary nodules*

	Anxiety	Non‐anxiety	*z*‐Value	*p*‐Value
Item 1 (Anxious mood)	3.52 ± 0.52	1.86 ± 0.27	−14.589	<0.001
Item 2 (Tension)	3.57 ± 0.53	1.87 ± 0.27	−14.700	<0.001
Item 3 (Fears)	3.42 ± 0.70	1.71 ± 0.28	−14.229	<0.001
Item 4 (Insomnia)	1.95 ± 0.012	0.27 ± 0.02	−15.915	<0.001
Item 5 (Intellectual)	0 ± 0.000	0.01 ± 0.003	−0.664	0.507
Item 6 (Depressed mood)	0.84 ± 0.113	0.09 ± 0.011	−11.647	<0.001
Item 7 (Somatic [muscular])	0.01 ± 0.011	0 ± 0.000	−3.019	0.03
Item 8 (Somatic [sensory])	0.19 ± 0.062	0.01 ± 0.004	−7.127	<0.001
Item 9 (Cardiovascular symptoms)	0.00 ± 0.00	0.00 ± 0.003	−0.574	0.566
Item 10 (Respiratory symptoms)	0.70 ± 0.094	0.08 ± 0.011	−11.565	<0.001
Item 11 (Gastrointestinal symptoms)	0.03 ± 0.034	0.00 ± 0.003	−1.020	0.308
Item 12 (Genitourinary symptoms)	0.00 ± 0.000	0.00 ± 0.000	0.000	1.000
Item 13 (Autonomic symptoms)	0.02 ± 0.023	0.00 ± 0.001	−1.904	0.057
Item 14 (Behaviour at interview)	3.39 ± 0.057	1.67 ± 0.031	−14.287	<0.001
Sum of psychic anxiety	16.69 ± 2.46	7.47 ± 3.39	−15.313	<0.001
Sum of somatic anxiety	0.97 ± 1.10	0.10 ± 0.38	−13.137	<0.001

^*^ Data are mean ± SD.

## 4 DISCUSSION

In the current study, the occurrence of anxiety in patients with unconfirmed pulmonary nodules is 9.89%, and the psychic anxiety was patients' predominant manifestation. Gender, respiratory symptoms, family history of malignant tumour and diameter of pulmonary nodules were independent influencing factors of anxiety.

It is widely recommended to screen lung cancer using LDCT for the high‐risk adults^9^ that might decrease lung cancer mortality by 20% and overall mortality by 6.7%.^7^ However, the vast majority of lung nodules most are false‐positive, individuals with which cannot benefit from the detection and may experience psychological harm as a result of a ‘near‐cancer’ diagnosis.^13^, ^19^ Even more patients will be found to have pulmonary nodules, as increasing people performed lung cancer screening annually. Anxiety is one of the most common psychological problems and is characterized by feelings of worry strong enough to interfere with daily activities.^20^ Within the category of anxiety disorders, DSM‐5 includes separation anxiety disorder, selective mutism, social anxiety disorder, panic disorder, agoraphobia, generalized anxiety disorder, substance/medication‐induced anxiety disorder and anxiety disorder due to another medical condition.^21^ However, the frequency, influencing factors and characteristics of lung cancer screening‐related psychological burden are still controversial and unclear. The NLST HRQoL study participants who were more likely to be female, white, more educated and unmarried compared with control participants.^16^ The psychological burden was mainly assessed by HRQoL, Spielberger State Trait Anxiety Inventory (STAI Form Y‐1)^16^, generic HRQoL (the 12‐item Short Form, SF‐12; the European Quality of Life questionnaire, EQ‐5D), the Spielberger State–Trait Anxiety Inventory (STAI‐6), lung‐cancer‐specific distress (the Impact of Event Scale, IES),^22^, ^23^ Hospital anxiety and depression scale (HAD) and GAD‐7.^13^ Except for the above mentioned questionnaires, Consequences of Screening‐Lung Cancer (COS‐LC)^24^, ^25^and Consequences Of Screening (COS)^25^ are also used to assess psychological burden, which have been developed and validated in the NELSON trial and in the Danish Lung Cancer Screening Trial (DLCST). This is the first research on anxiety assessment of patients with pulmonary nodules via HAMA. HAMA is considered one of the most widely used rating scales in clinical trials and occupational health‐related studies and as a monitoring tool in consulting room, with reasonable inter‐rater reliability and good 1‐week retest reliability.^26^

In the current study, only 38.54% patients were defined as absence of anxiety. Moreover, as high as 61.45% patients with unconfirmed pulmonary nodules exist various severity of anxiety, whereas vast majority patients (51.57%) were rated as mild or probable anxiety with a score between 8 to 14. Hence, the occurrence of anxiety is 9.89% according to that the anxiety group is defined by sum score >14, which is higher than that of anxiety disorders in China,^27^ with a prevalence of 5.0%. The occurrence of anxiety in patients with pulmonary nodules in this study is lower than 59.3%^13^ and 39.8%^14^ reported before, which may be related to population differences and different scales used for evaluation. Li et al.^13^ reported that the frequency of visits and social support had significant effects on anxiety, and specially social support was an independent influencing factor of anxiety; however gender and symptoms were not influencing factors of anxiety. In contrast, in this study, logistic regression analysis showed that gender, respiratory symptoms, family history of malignant tumour and diameter of pulmonary nodules were independent influencing factors of anxiety. Similarly, scores of HAMA and structural factors (psychic anxiety and somatic anxiety) were significantly higher in female than in male patients with irritable bowel syndrome (*p* < 0.01).^28^ Female was a significant predictor of anxiety among patients undergoing elective percutaneous transluminal coronary angioplasty,^29^ colonoscopy,^30^ dental treatment,^31^ and so on. Therefore, women seem to be more prone to this kind of anxiety related to diagnosis and treatment. And this study found for the first time that the diameter of pulmonary nodules was also one of the independent risk factors for anxiety, which may be related to the increase in knowledge about lung nodules among patients. After all, the guides recommended that nodules >8 mm need to be taken serious.

In addition, this study conducted a further analysis of the characteristics of pulmonary nodules anxiety for the first time. HAMA scale is consist of somatic anxiety and psychic anxiety. In the anxiety group, 6 items (1, 2, 3, 4, 6 and 14) of psychic anxiety was significantly higher than non‐anxiety, and only 3 items (7, 8 and 10) of somatic anxiety was significantly higher than non‐anxiety. Further analysis of psychic and somatic anxiety in 88 patients with anxiety showed sum of psychic anxiety was significantly higher than somatic anxiety. So, our study revealed that the anxiety of patients with pulmonary nodules is mainly manifested as psychic anxiety. Previous research showed individuals with major depressive disorder scoring high on ‘psychic anxiety’ had elevated F2‐isoprostanes, and this was not seen with ‘depressed mood’ scores.^32^ Zeng et al.^33^ reported that electroacupuncture helps to improve HAMA Psychic Anxiety subscale in methamphetamine addicts during abstinence. Another research indicated statistically significant improvements with vilazodone were found on HAMA Psychic Anxiety subscale, except the HAMA Somatic Anxiety subscale.^34^ Therefore, research of the characteristics of pulmonary nodules anxiety may help to furtherly explore specific biomarkers and treatment methods.

## 5 CONCLUSION

Vast majority of patients with unconfirmed pulmonary nodules were found to suffer various severity of anxiety in the current study, which was mainly manifested as psychic anxiety. And gender, respiratory symptoms, family history of malignant tumour and diameter of pulmonary nodules were independent influencing factors of anxiety. Effective strategies of communication and management are urgently need to been explored and provided for improving the mental health.

## CONFLICT OF INTEREST

The authors declare no conflict of interest.

## ETHICS STATEMENT

The informed consent of all participants was obtained. The institutional review board of the First Affiliated Hospital of Chongqing Medical University Ethics Board approved the study, code 2019–083.

## AUTHOR CONTRIBUTIONS

Xiao‐Hui Wang, and Li Yang, made substantial contributions to conceive and design the study. Xiao‐Hui Wang, was in charge of the manuscript draft. Xiao‐Hui Wang, Ting Wang and Min Ao collected and confirmed data accuracy. Ting Wang, and Li Yang, applied for the ethical approval. Jinglan He and Long‐Biao Cui, as psychiatrists, were responsible for training psychological scale evaluation and consultation. Xiao‐Hui Wang and Jun Duan were responsible for statistical analysis of data. All authors made substantial revisions to the manuscript.

## Data Availability

The data that support the findings of this study are available from the corresponding author upon reasonable request.

## References

[1] Cao M , Chen W . Epidemiology of lung cancer in China. Thorac Cancer. 2019;10(1):3‐7. doi:10.1111/1759-7714.12916 30485694 PMC6312841

[2] Chen W , Zhang S , Zou X . Evaluation on the incidence, mortality and tendency of lung cancer in China. Thorac Cancer. 2010;1(1):35‐40. doi:10.1111/j.1759-7714.2010.00011.x 27755783

[3] Tseng CH , Tsuang BJ , Chiang CJ , et al. The relationship between air pollution and lung cancer in nonsmokers in Taiwan. J Thorac Oncol. 2019;14(5):784‐792. doi:10.1016/j.jtho.2018.12.033 30664991

[4] De Matteis S , Consonni D , Lubin JH , et al. Impact of occupational carcinogens on lung cancer risk in a general population. Int J Epidemiol. 2012;41(3):711‐721. doi:10.1093/ije/dys042 22467291 PMC3396321

[5] Who . WHO report on the global tobacco epidemic 2019: offer help to quit tobacco use. 2019.

[6] Church TR , Black WC , Aberle DR , et al. Results of initial low‐dose computed tomographic screening for lung cancer. N Engl J Med. 2013;368(21):1980‐1991. doi:10.1056/NEJMoa1209120 23697514 PMC3762603

[7] Aberle DR , Adams AM , Berg CD , et al. Reduced lung‐cancer mortality with low‐dose computed tomographic screening. N Engl J Med. 2011;365(5):395‐409. doi:10.1056/NEJMoa1102873 21714641 PMC4356534

[8] Fan L , Wang Y , Zhou Y , et al. Lung cancer screening with low‐dose CT: baseline screening results in Shanghai. Acad Radiol. 2019;26(10):1283‐1291. doi:10.1016/j.acra.2018.12.002 30554839

[9] Zhou Q , Fan Y , Wang Y , et al. China National Lung Cancer Screening Guideline with low‐dose computed tomography (2018 version). Zhongguo Fei Ai Za Zhi. 2018;21(2):67‐75. doi:10.3779/j.issn.1009-3419.2018.02.01 29526173 PMC5973012

[10] Du Jia HMQH . Results of lung cancer screening among urban residents in Chongqing, 2012~2017. China Cancer. 2018;27(5):328‐332.

[11] Gu Xiuying GXZJ . Analysis of lung cancer screening results of 9265 urban residents in Urumqi from year 2014 to 2016. Pract Oncol J. 2017;3(31):242‐245.

[12] Freiman MR , Clark JA , Slatore CG , et al. Patients' knowledge, beliefs, and distress associated with detection and evaluation of incidental pulmonary nodules for cancer: results from a multicenter survey. J Thorac Oncol. 2016;11(5):700‐708. doi:10.1016/j.jtho.2016.01.018 26961390 PMC4851914

[13] Li L , Zhao Y , Li H . Assessment of anxiety and depression in patients with incidental pulmonary nodules and analysis of its related impact factors. Thorac Cancer. 2020;11(6):1433‐1442. doi:10.1111/1759-7714.13406 32212379 PMC7262923

[14] Wang L , Wei Y , Hu H , Zhang X , Zheng M , Fei G . Correlation between anxiety, depression and changes in Th17/Treg and inflammatory levels in patients with pulmonary nodules. Zhongguo Fei Ai Za Zhi. 2020;23(7):554‐560. doi:10.3779/j.issn.1009-3419.2020.102.30 32702789 PMC7406438

[15] Clark ME , Bedford LE , Young B , et al. Lung cancer CT screening: psychological responses in the presence and absence of pulmonary nodules. Lung Cancer. 2018;124:160‐167. doi:10.1016/j.lungcan.2018.08.001 30268456

[16] Gareen IF , Duan F , Greco EM , et al. Impact of lung cancer screening results on participant health‐related quality of life and state anxiety in the National Lung Screening Trial. Cancer. 2014;120(21):3401‐3409. doi:10.1002/cncr.28833 25065710 PMC4205265

[17] Zimmerman M , Thompson JS , Diehl JM , Balling C , Kiefer R . Is the DSM‐5 anxious distress specifier interview a valid measure of anxiety in patients with generalized anxiety disorder: a comparison to the Hamilton anxiety scale. Psychiatry Res. 2020;286:112859. doi:10.1016/j.psychres.2020.112859 32088508

[18] Hamilton M . The assessment of anxiety states by rating. Br J Med Psychol. 1959;32(1):50‐55. doi:10.1111/j.2044-8341.1959.tb00467.x 13638508

[19] Slatore CG , Wiener RS . Pulmonary nodules: a small problem for many, severe distress for some, and how to communicate about it. Chest. 2018;153(4):1004‐1015. doi:10.1016/j.chest.2017.10.013 29066390 PMC5989642

[20] Schuyler D . When to refer to palliative care. Prim Care Companion CNS Disord. 2016;18(6):27015. doi:10.4088/PCC.16f02074 28033458

[21] Kalin NH . Novel insights into pathological anxiety and anxiety‐related disorders. Am J Psychiatry. 2020;177(3):187‐189. doi:10.1176/appi.ajp.2020.20010057 32114781

[22] van den Bergh KA , Essink‐Bot ML , Borsboom GJ , et al. Short‐term health‐related quality of life consequences in a lung cancer CT screening trial (NELSON). Br J Cancer. 2010;102(1):27‐34. doi:10.1038/sj.bjc.6605459 19935789 PMC2813757

[23] van den Bergh KA , Essink‐Bot ML , Borsboom GJ , Scholten ET , van Klaveren RJ , de Koning HJ . Long‐term effects of lung cancer computed tomography screening on health‐related quality of life: the NELSON trial. Eur Respir J. 2011;38(1):154‐161. doi:10.1183/09031936.00123410 21148229

[24] van den Bergh KA , Essink‐Bot ML , van Klaveren RJ , de Koning HJ . Informed decision making does not affect health‐related quality of life in lung cancer screening (NELSON trial). Eur J Cancer. 2010;46(18):3300‐3306. doi:10.1016/j.ejca.2010.05.030 20580546

[25] Aggestrup LM , Hestbech MS , Siersma V , Pedersen JH , Brodersen J . Psychosocial consequences of allocation to lung cancer screening: a randomised controlled trial. BMJ Open. 2012;2(2):e663. doi:10.1136/bmjopen-2011-000663 PMC3293139 22382119

[26] Thompson E . Hamilton rating scale for anxiety (HAM‐A). Occup Med (Lond). 2015;65(7):601. doi:10.1093/occmed/kqv054 26370845

[27] Huang Y , Wang Y , Wang H , et al. Prevalence of mental disorders in China: a cross‐sectional epidemiological study. Lancet Psychiatry. 2019;6(3):211‐224. doi:10.1016/S2215-0366(18)30511-X 30792114

[28] Zhu L , Huang D , Shi L , et al. Intestinal symptoms and psychological factors jointly affect quality of life of patients with irritable bowel syndrome with diarrhea. Health Qual Life Outcomes. 2015;13(1):49. doi:10.1186/s12955-015-0243-3 PMC4414422 25925746

[29] Astin F , Jones K , Thompson DR . Prevalence and patterns of anxiety and depression in patients undergoing elective percutaneous transluminal coronary angioplasty. Heart Lung. 2005;34(6):393‐401. doi:10.1016/j.hrtlng.2005.05.002 16324958

[30] Baudet JS , Aguirre‐Jaime A . The sedation increases the acceptance of repeat colonoscopies. Eur J Gastroenterol Hepatol. 2012;24(7):775‐780. doi:10.1097/MEG.0b013e32835376a2 22522140

[31] Yuzugullu B , Gulsahi A , Celik C , Bulut S . Dental anxiety and fear: relationship with oral health behavior in a Turkish population. Int J Prosthodont. 2014;27(1):50‐53. doi:10.11607/ijp.3708 24392477

[32] Steenkamp LR , Hough CM , Reus VI , et al. Severity of anxiety—but not depression—is associated with oxidative stress in major depressive disorder. J Affect Disord. 2017;219:193‐200. doi:10.1016/j.jad.2017.04.042 PMC5550320 28564628

[33] Zeng L , Tao Y , Hou W , Zong L , Yu L . Electro‐acupuncture improves psychiatric symptoms, anxiety and depression in methamphetamine addicts during abstinence: a randomized controlled trial. Medicine (Baltimore). 2018;97(34):e11905. doi:10.1097/MD.0000000000011905 PMC6112927 30142795

[34] Thase ME , Chen D , Edwards J , Ruth A . Efficacy of vilazodone on anxiety symptoms in patients with major depressive disorder. Int Clin Psychopharmacol. 2014;29(6):351‐356. doi:10.1097/YIC.0000000000000045 PMC4186734 24978955

